# Retraction Note: Modulatory effect of liraglutide on doxorubicin-induced testicular toxicity and behavioral abnormalities in rats: role of testicular-brain axis

**DOI:** 10.1007/s00210-025-04046-6

**Published:** 2025-03-19

**Authors:** Shorouk A. Alafifi, Sara A. Wahdan, Alzahraa A. Elhemiely, Doaa A. Elsherbiny, Samar S. Azab

**Affiliations:** 1 Pharmacology & Toxicology Department, Faculty of Pharmacy, Ain Shams University, Cairo, 11566 Egypt; 2 Researcher of Pharmacology, Egyptian Drug Authority, Cairo, Egypt

**Retraction Note: Naunyn-Schmiedeberg's Archives of Pharmacology (2023) 396:2987–3005**

10.1007/s00210-023-02504-7

The Editor-in-Chief has retracted this article. Concerns were raised about figure 2 and the use of the same images at different magnification with inappropriate scale bars. When the images were re-sized to match the different magnifications, the scale bars were not proportional, which calls into question the veracity of this figure.

The authors were not able to provide a satisfactory explanation or replacement figure that would address the concerns. Therefore, the Editor has lost confidence in the data and conclusions of this article.

Authors Sara A. Wahdan and Samar S. Azab disagree with this retraction. Author Doaa A. Elsherbiny and Shorouk A. Alafifi did not respond to correspondence from the Publisher about this retraction. Author Alzahraa A. Elhemiely could not be contacted by the publisher.

